# Darwin’s Duchenne: Eye Constriction during Infant Joy and Distress

**DOI:** 10.1371/journal.pone.0080161

**Published:** 2013-11-20

**Authors:** Whitney I. Mattson, Jeffrey F. Cohn, Mohammad H. Mahoor, Devon N. Gangi, Daniel S. Messinger

**Affiliations:** 1 Department of Psychology, University of Miami, Miami, Florida, United States of America; 2 Department of Psychology, University of Pittsburgh, Pittsburgh, Pennsylvania, United States of America; 3 Department of Computer Science, Carnegie Mellon University, Pittsburgh, Pennsylvania, United States of America; 4 Department of Electrical and Computer Engineering, University of Denver, Denver, Colorado, United States of America; 5 Department of Pediatrics, University of Miami, Miami, Florida, United States of America; 6 Department of Electrical and Computer Engineering, University of Miami, Miami, Florida, United States of America; University of Milan, Italy

## Abstract

Darwin proposed that smiles with eye constriction (Duchenne smiles) index strong positive emotion in infants, while cry-faces with eye constriction index strong negative emotion. Research has supported Darwin’s proposal with respect to smiling, but there has been little parallel research on cry-faces (open-mouth expressions with lateral lip stretching). To investigate the possibility that eye constriction indexes the affective intensity of positive and negative emotions, we first conducted the Face-to-Face/Still-Face (FFSF) procedure at 6 months. In the FFSF, three minutes of naturalistic infant-parent play interaction (which elicits more smiles than cry-faces) are followed by two minutes in which the parent holds an unresponsive still-face (which elicits more cry-faces than smiles). Consistent with Darwin’s proposal, eye constriction was associated with stronger smiling and with stronger cry-faces. In addition, the proportion of smiles with eye constriction was higher during the positive-emotion eliciting play episode than during the still-face. In parallel, the proportion of cry-faces with eye constriction was higher during the negative-emotion eliciting still-face than during play. These results are consonant with the hypothesis that eye constriction indexes the affective intensity of both positive and negative facial configurations. A preponderance of eye constriction during cry-faces was observed in a second elicitor of intense negative emotion, vaccination injections, at both 6 and 12 months of age. The results support the existence of a Duchenne distress expression that parallels the more well-known Duchenne smile. This suggests that eye constriction–the Duchenne marker–has a systematic association with early facial expressions of intense negative and positive emotion.

## Introduction

Following early research by French neurologist Duchenne de Boulogne, Darwin highlighted the role of eye constriction (*orbicularis oculi, pars orbitalis*) in facial expressions of positive emotion [1], [2]. Subsequent research has confirmed that smiles with eye constriction (Duchenne smiles) are indices of strong positive emotion. In both infants and adults, Duchenne smiles are a more frequent response to positive emotion elicitors–and perceived as more joyful–than other smiles [3], [4], [5], [6], [7], [8], [9].

Darwin also proposed that eye constriction plays a central role in weeping and crying, particularly during infancy [1]. Infants have historically provided a window with which to understand the ontogeny and dynamics of facial expressions [10], [11]. Cry-faces are the prototypic expression of negative emotion in infancy [12], [13], [14]. Like smiles, cry-faces may or may not be accompanied by eye constriction [15], [16], [17]. The presence of eye constriction in cry-faces has been documented in response to pain in infants [18], [19], [20] and adults [21], [22]. However, there have been no simultaneous examinations of the role of infant eye constriction in smiling and cry-face expressions as reactions to experimental elicitors of positive and negative emotion.

### Study 1

In study 1, we examined whether the Duchenne marker, eye constriction, indexes the emotional intensity of both positive *and* negative infant facial expressions. To do so, we utilized an experimental manipulation of parent responsivity, the Face-to-Face/Still-Face (FFSF) [23], [24], [25]. In the FFSF, the parent first plays with the infant (Play) and then becomes expressionless and unresponsive (Still-Face). Play elicits more positive emotion (i.e. higher proportions of infant smiling) than the Still-Face; the Still-Face elicits more negative emotion (i.e. a higher proportion of cry-face expressions) than Play. Based on these findings, we reasoned that smiles during Play would be more emotionally positive than smiles in the Still-Face, and that cry-faces in the Still-Face would be more emotionally negative than cry-faces during Play. Accordingly, we hypothesized that a greater proportion of smiles would involve eye constriction during Play than during the Still-Face; and that a greater proportion of cry-faces would involve eye constriction during the Still-Face than during Play.

More generally, if the Duchenne marker, eye constriction, indexes intense emotion, it should be associated with the strength of accompanying smiles and cry-faces. There does not appear to be evidence that the *presence* of the Duchenne marker is associated with either stronger smiles or stronger cry-faces in infants. In the current dataset and others [26], however, the strength of eye constriction covaries with the strength of smiles and cry-faces [9]. Consequently, we expected the Duchenne marker to be associated with both stronger smiles and cry-faces.

### Study 2

Cry-face expressions and eye constriction have been found to accompany infant facial expressions of pain. Through 12 months, infant cry-face expressions in response to injections frequently involve eye constriction produced by *orbicularis oculi pars orbitalis* (AU6) and/or eye shutting *orbicularis oculi pars palpebralis* (AU7) [18], [19], [27]. However, the specific role of eye constriction–AU6, the marker associated with Duchenne smiling–has not been assessed in cry-face expressions. Moreover, the likelihood of eye constriction and cry-faces co-occurring, and possible changes in this correspondence over developmental time, have not been assessed. To address this gap, Study 2 investigated whether eye constriction was involved in infants’ cry-face expressions using a naturalistic elicitor of pain, which is associated with intense negative emotion. Video of vaccination injections at two ages–6 and 12 months–were obtained from publicly available recordings [28]. These recordings were used to ascertain the likelihood of cry-faces involving eye constriction, and to determine whether this co-occurrence changed with age.

## Methods

### Study 1

#### Participants

Twelve six-month-olds and their parents (11 mothers, 1 father) were video-recorded in the FFSF [9] to elicit a range of negative and positive infant emotional expressions. Play lasted three minutes and the Still-Face lasted two minutes. The six-month-olds (*M* = 6.20, *SD* = 0.43) were 66.7% male and ethnically diverse (16.7% African American; 16.7% Asian American, 33.3% Hispanic American, and 33.3% European American).

#### Facial Coding

Facial expressions were coded using Action Units (AU) of the anatomically-based Facial Action Coding System (FACS) [15]. Smiles were indexed by oblique action of *zygomaticus major* (AU12), cry-faces by lateral action of *risorius* (AU20), and eye constriction by the action of *orbicularis oculi, pars orbitalis* (AU6), which draws the cheeks and skin surrounding the temples toward the eyes. Coding of these AUs was automated [9]. Active appearance and shape models (AAM) tracked rigid and non-rigid facial features over contiguous video frames [29]. Shape features of the face were represented as relations between 66 (x, y) points whose motion was normalized to control for rigid head motion. Appearance was represented as the grayscale value of each pixel in the normalized face shape model. Shape and appearance features was submitted to Laplacian data reduction to produce a set of 29 features per video frame [30]. These features were input for separate support vector machines (SVM) [31], [32].

SVM classifiers were trained with manual FACS coding using a leave-one-out cross-validation procedure to classify the intensity of AUs. Intensity ranged from “trace” (A) to “maximal” (E) for each AU. Automated coding exhibited high inter-system concordance with manual coding of intensity [9]. We next dichotomized intensity codes to capture the presence (FACS B “slight” intensity of greater) [15] or absence of each AU to focus on the role of the Duchenne marker. Automated measurement and manual measurement showed high reliability on this measure (mean Cohen’s Kappa, which accounts for chance agreement, was.78 for eye constriction,.77 for smiles, and.76 for cry-faces).

#### Ethics statement

Participants’ parents provided written informed consent and all procedures were approved by the University of Miami Institutional Review Board.

### Study 2

#### Participants

Videos of regularly scheduled infant vaccinations were located on http://www.youtube.com [33], using a key word search for “Baby,” “Shots,” and either “6 months” or “12 months.” Based on both video title and audio reports during the video, 12 infants were identified as 6 months of age and 12 infants were identified as 12 months of age. Videos were downloaded and edited to include the ten seconds following the first injection recorded.

#### Facial coding

As in Study 1, cry-faces were indexed by AU20 and eye constriction by AU6. Cry-faces and eye constriction were coded manually on a frame-by-frame basis for presence (FACS B “slight” intensity or greater) or absence. Inter-rater reliability was high (mean Kappa was.86 for eye constriction and.71 for cry-faces).

#### Ethics statement

Videos of vaccinations were gathered from a publicly available listing.

## Results

### Study 1

**Figure 1:**
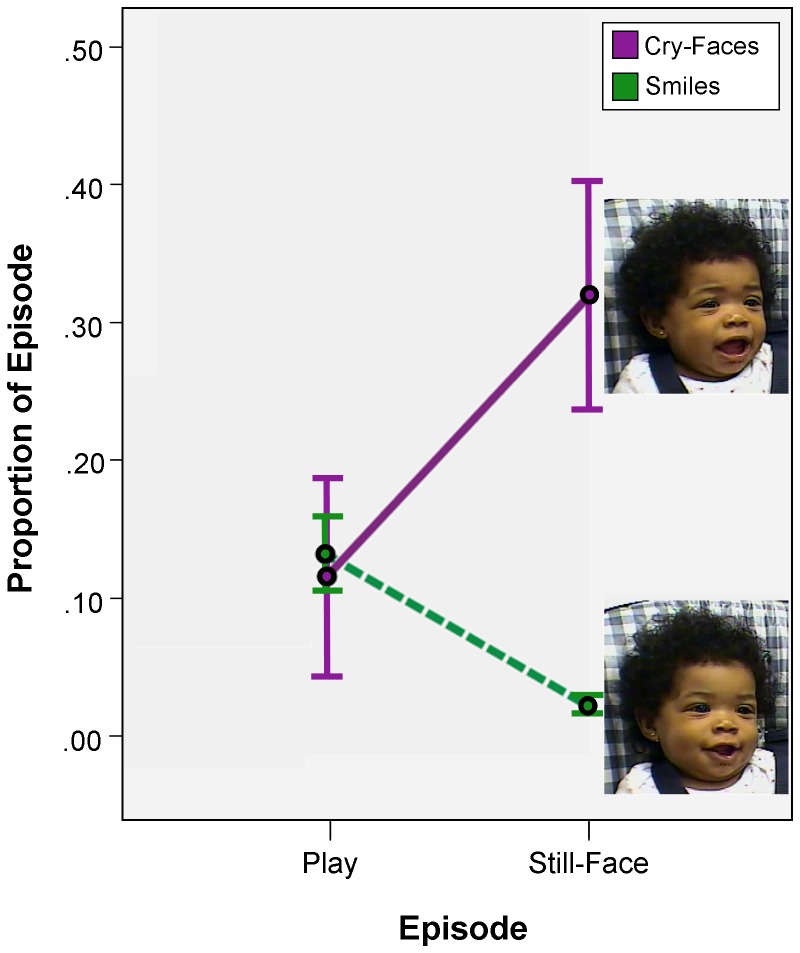
Time in smiling and cry-faces as a proportion of time in each episode of the Face-to-Face/Still-Face (FFSF). Error bars indicate standard errors of the mean. The images are of smiles and of cry-faces without eye constriction. The images are from a six-month-old in the FFSF in the current study. Written informed consent, as outlined in the PLOS consent form, was obtained for publication of these images.

We posited that eye constriction would be differentially distributed with smiles and cry-faces during the FFSF. To lay the groundwork for testing this hypothesis, we used repeated-measures ANOVAs to ascertain whether there were still-face effects in the *overall* levels of smiles and cry-faces in the FFSF. Smiles and cry-faces were distributed differentially in the Play and Still-Face episodes of the FFSF, *F* (2, 22) = 7.24, *p*<.01, *η_p_^2^* = .40 (see Figure 1). The mean proportion of time involving smiling declined from Play (*M* = .13, *SD* = .08) to the Still-Face (*M* = .02, *SD* = .03), *F* (1, 11) = 17.10, *p*<.01, *η_p_^2^* = .61. The mean proportion of time involving cry-faces increased from Play (*M* = .11, *SD* = .25) to the Still-Face (*M* = .32, *SD* = .29), *F* (1, 11) = 4.97, *p*<.05, *η_p_^2^* = .31. These overall still-face effects are the background against which we test whether the proportion of smiles involving eye constriction and the proportion of cry-faces involving eye constriction vary systematically over the course of the FFSF.

**Figure 2:**
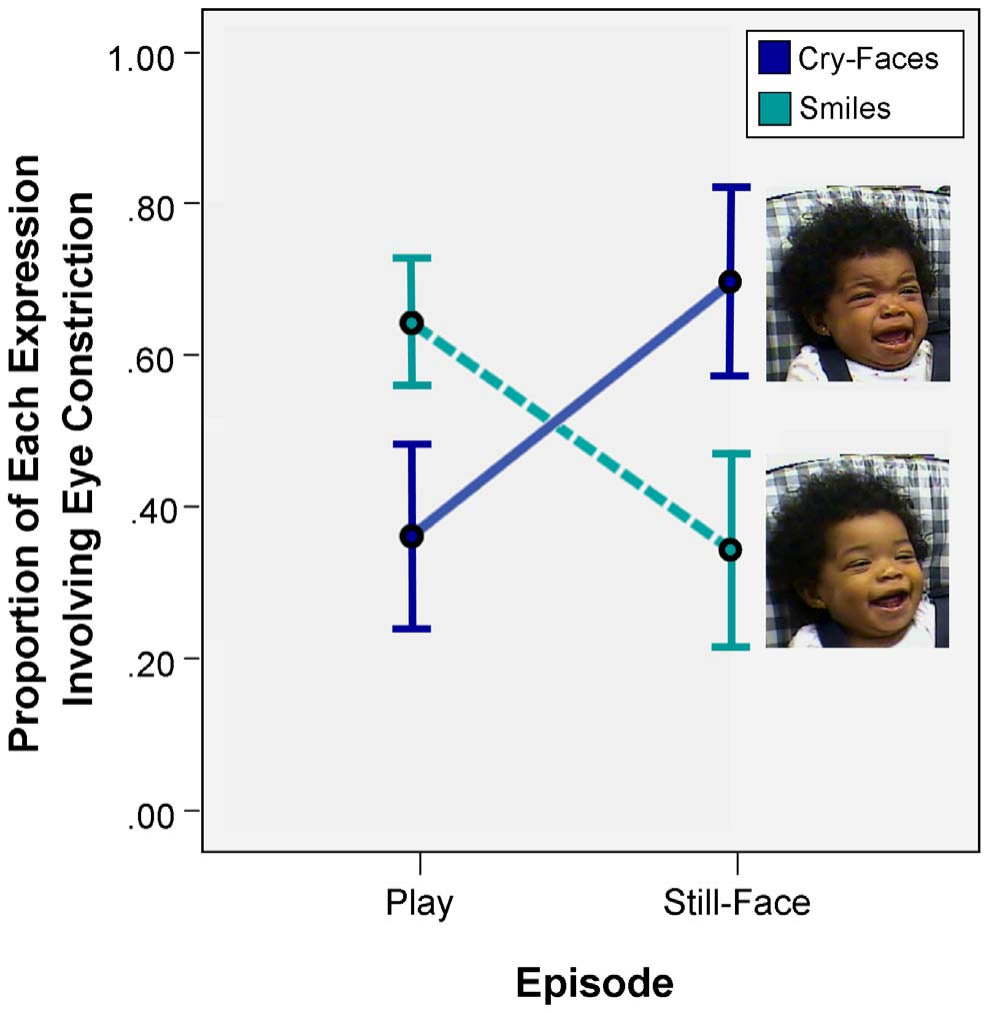
Eye constriction (the Duchenne marker) is differentially associated with smiles and cry-faces in the Face-to-Face/Still-Face (FFSF). Mean proportions of smiles and of cry-faces occurring with eye constriction. Error bars indicate standard errors of the mean. The images are of smiles and cry-faces with eye constriction. The images are from a six-month-old in the FFSF in the current study. Written informed consent, as outlined in the PLOS consent form, was obtained for publication of these images.

A repeated-measures ANOVA indicated that proportions of smiling and cry-faces that involved eye constriction were differentially distributed over episodes of the FFSF, *F* (2, 22) = 11.28, *p*<.001, *η_p_^2^* = .51 (see Figure 2). A higher proportion of smiles involved eye constriction during Play (*M* = .64, *SD* = .30) than did smiles during the Still-Face (*M* = .34, *SD* = .38), *F* (1, 11) = 6.70, *p* = .03, *η_p_^2^* = .38. A higher proportion of cry-faces involved eye constriction during the Still-Face (*M* = .70, *SD* = .38) than during Play (*M* = .36, *SD* = .39), *F* (1, 11) = 11.43, *p*<.01, *η_p_^2^* = .51. These results provide evidence in support of the hypothesis. In a positive-emotion eliciting context, smiling was more likely to be accompanied by eye constriction. In a negative-emotion eliciting context, cry-faces were more likely to be accompanied by eye constriction.

We next asked whether the presence of eye constriction was associated with stronger smiles and cry-faces. Smiles involving eye constriction were significantly stronger (*M* = 3.07, *SD* = .21) than smiles without eye constriction (*M* = 2.61, *SD* = .33), *t*(10) = 4.86, *p* = .001. In parallel fashion, cry-faces involving eye constriction were significantly stronger (*M* = 3.61, *SD* = .38) than cry-faces without eye constriction (*M* = 3.16, *SD* = .26), *t*(9) = 3.77, *p*<.01. These results indicate that the Duchenne marker, eye constriction, is associated with the intensity of both smiles and cry-faces, indices of positive and negative emotion, respectively.

### Study 2

We determined the overall proportion of time involving cry-face expressions after injections and tested whether this proportion varied with age. The mean proportion of time involving cry-faces was.50. There was not a significant difference in the proportion of time involving cry-faces at 6 months (*M* = .43, *SD* = .25) compared to 12 months (*M* = .56, *SD* = .24), *F* (1, 22) = 1.71, *p* = .20. We next examined the proportion of time in which cry-faces were accompanied by eye constriction. The mean proportion of time in cry-faces which involved eye constriction was.87. There were no differences in the proportion of cry-faces involving eye constriction at 6 (*M* = .82, *SD* = .28) and 12 months (*M* = .92, *SD* = .07), *F* (1, 21) = 1.43, *p* = .24. These results indicate that in an intense negative-emotion eliciting context, cry-faces were likely to be accompanied by eye constriction at both 6 and 12 months of age. That is, the overwhelming majority of cry-faces occurring in response to a painful elicitor of negative emotion involved the Duchenne marker.

## Discussion

### Study 1

Experimental evidence supported Darwin’s supposition that eye constriction would be associated with more emotionally positive smiles and more emotionally negative cry-faces. Smiling during the face-to-face play with the parent, which was intended to elicit positive emotion, involved a higher proportion of smiling with eye constriction than smiling during the Still-Face. The Still-Face, intended to elicit negative emotion, involved a higher proportion of cry-faces with eye constriction than cry-faces that occurred during face-to-face play. The results indicate that when infants are engaged in play with a parent, their smiles are more emotionally positive than when they are trying to elicit a response from a non-responsive parent. Likewise, when infants are stymied by their non-responsive parent, their distress expressions are more emotionally negative than distress expressions that occur during play. Finally, the Duchenne marker, eye constriction, accompanied both stronger smiles and stronger cry-faces.

Study 1 results add to a growing body of research suggesting that smiling with eye constriction is stronger and more likely to occur in situations that elicit positive emotion than smiles without eye constriction [5], [6], [34]. Early distress expressions that involved eye constriction also tended to be stronger and were more likely to occur in periods intended to elicit negative emotion than distress expressions without eye constriction. To ascertain the generality with which infants respond to strong elicitors of negative emotion with cry-faces involving eye constriction, we next examined responses to naturally occurring vaccination injections.

### Study 2

Study 2 vaccination findings extend the Study 1 FFSF cry-face results to a naturalistic elicitor of intense negative emotion. Immediately following vaccination injections, the overwhelming majority of cry-faces involved eye constriction at both 6 and 12 months. These results provide an anatomically specific documentation of infant eye constriction responses to painful injections [18], [27]. In response to this elicitor of intense negative emotion, infants combined a cry-face expression with eye constriction, the same Duchenne marker they combine with smiling during the elicitation of intense positive emotion during play. These cry-faces involving eye constriction, termed Duchenne distress expressions (see Fridlund) [35], were the predominant responses both to parents’ abruptly ceasing playful interaction and to a noxious stimulus.

## General Discussion

Together, Studies 1 and 2 support the contention that Duchenne smiling indexes strong positive emotion [4], [5], [6], [7] and suggest the existence of an infant Duchenne distress expression, which indexes strong negative emotion. These results are a generalization to negative expressions of the original insight that Duchenne smiling indexed strong positive emotion [2], [4]. Specifically, eye constriction’s role in indexing emotional intensity in two central infant facial expressions suggests parsimony in the early communication of emotion. Ultimately these findings support the contention of Darwin and others that a given facial action may have a consistent function in a variety of facial expressions [36], [37], [38].

Eye constriction (*obicularis occuli pars orbitalis*), the Duchenne marker, reduces but does not completely occlude the visual field. This suggests that the Duchenne marker may regulate exposure to intense emotional stimuli [34], and may also increase the expresser’s attention to his or her own internal emotional state [8]. Duchenne smiling and Duchenne distress appear to serve, respectively, to communicate intense positive engagement and an intense need for comfort. Smiling and the cry-face expression are the infant’s most commonly used facial configurations, suggesting the importance of eye constriction to early emotion expression and communication [12], [13].

In adults, the role of eye constriction is well documented in Duchenne smiling [3], [4], [7]. Eye constriction is also a key element of the adult pain configuration [21], [22], and may index the intensity of this expression which, when it is maximally displayed, is an adult analog of the infant cry-face. The same expressive configuration is present during other intense experiences such as orgasm [39], suggesting that eye constriction is associated with the intensity of facial expressions of extreme positive and negative valence in adults.

From an evolutionary perspective, eye constriction provides a parsimonious means for indexing the intensity of both positive and negative emotions. However, an evolutionary focus on the function of facial movements for the organism in its environment suggests that eye constriction will not have an identical role in all (adult) expressions of negative emotion [22]. Facial expressions of fear, for example, may serve to enhance sensory input, widening the visual field to facilitate quick defensive reactions [40]. Anger configurations in adults also involve eye opening (AU5) [15], which is likely to facilitate and communicate potential aggression to the target [35], [41]. This suggests that the intensity of fear and anger might be indexed by eye opening rather than eye constriction. Disgust, by contrast, is characterized by rejecting sensory input through eye and nostril constriction [40]. Eye constriction might index the intensity of disgust, and of sadness, which may also involve a narrowing of the visual field in adults, but this remains a topic for future research. Ultimately, the current focus on the general functions of facial actions across a range of expressions (see Susskind et al.) [40] is likely to produce new insights into both expression-specific and pan-expression features of expressive action.

The face is one modality of emotional expression. Aviezer, Trope, and Todorov found that other modalities of expression such as body posture carry more weight than the face when adults rate the valence of still images of other adults during positive and negative events [42]. Nevertheless the facial expressions in response to both positive and negative events (e.g., winning or losing a match point during a tennis match) exhibited in Aviezer et al.’s figures (Figures 1–4) [42] all involved eye constriction and/or eye shutting, potentially underlining the role of these actions in communicating intense positive and negative emotional valence.

Ultimately, the degree to which facial and other modalities of bodily expression such as the voice provide consistent (or inconsistent) signals of affective state throughout the lifespan is a rich area of continued research. Two research strategies adopted here–automated measurement of expression and the analysis of naturally occurring emotional reactions in publicly shared repositories–are promising approaches to future multimodal explorations of emotional functioning. In the current report, these diverse research strategies highlight a common function of a specific facial action in indexing both intense positive and negative emotion.
